# Giant cardiac myxoma in a patient with thrombocytopenia: is there a physiopathologic link? (a case report)

**DOI:** 10.11604/pamj.2020.37.348.26109

**Published:** 2020-12-16

**Authors:** Taamallah Karima, Besbes Bouthaina, Haggui Abdeddayem, Fehri Wafa

**Affiliations:** 1 Department of Cardiology, Military Hospital of Tunis, Tunis, Tunisia

**Keywords:** Cardiac myxoma, cardiac surgery, echocardiography, thrombocytopenia, case report

## Abstract

Atrial myxoma is the most common primary cardiac tumor. We report the case of left atrium myxoma accompanied by severe thrombocytopenia in a 72-years-old woman. The thrombocytopenia has been discovered 5 years ago, it was explored, no obvious cause was found, the diagnosis of idiopathic thrombocytopenia was retained based on clinical and paraclinical arguments, corticosteroid treatment was ineffective and platelet count remains low. Complete surgical excision of the mass was performed. Platelet count was gradually increased to reach 95 103/µl after 6 months postoperatively. In this report, we highlight that thrombocytopenia might be one rare hematological manifestation of myxoma but need more cases for support. By illustrating this association, we hope to facilitate an earlier diagnosis of cardiac myxoma to treat and avoid complications of both thrombocytopenia and myxoma.

## Introduction

Cardiac myxoma is the most common primary cardiac tumor. Its clinical presentation is a wide range of symptom spectrum going from asymptomatic incidental masses to serious life-threatening cardiovascular complications. Echocardiography is still the gold standard tool for diagnosis as well as follow-up. To prevent embolization and sudden death occurring in up to one-third of patients, urgent surgical resection of myxomas is the accepted treatment [1]. Thrombocytopenia has very rarely been reported in association with myxoma. Syncope is also a rare manifestation of cardiac myxoma. We report the case of left atrium myxoma in a 72 years old woman, presenting with iterative episodes of fainting and syncope and thrombocytopenia.

## Patient and observation

**Figure 1:**
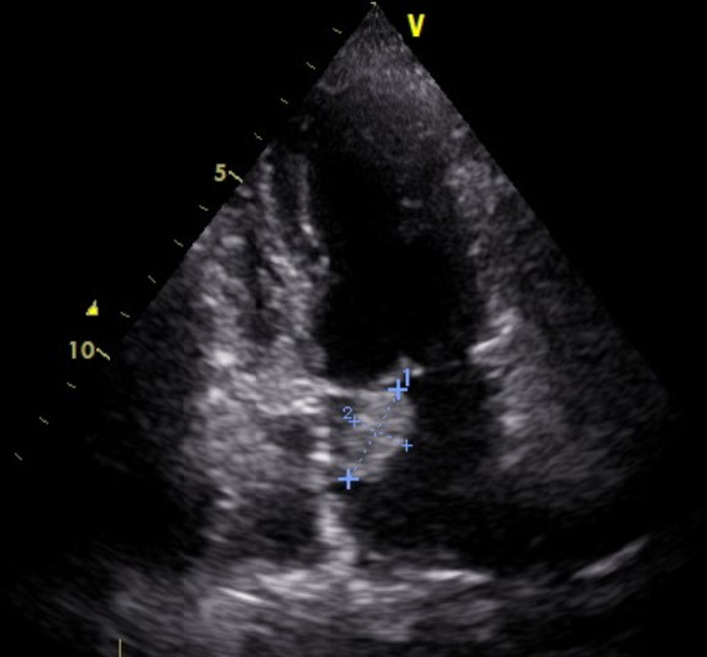
left atrial mass on transthoracic echocardiography (apical view)

A 72-years-old female presented with fatigue, iterative episodes of fainting with two episodes of exertional syncope evolving for the last 6 months. The patient had been followed for idiopathic thrombocytopenia diagnosed five years ago and treated with folic acid and corticosteroids. The idiopathic origin of this thrombocytopenia has been retained after having carried out a paraclinical assessment including negative viral serologies, a bone marrow biopsy confirming the peripheral origin of the thrombocytopenia by showing increased megakaryocytes, immunological laboratory tests that were normal, and an immunoelectrophoresis of protein that was unremarkable. No anemia, negative Coomb's test, normal lactate dehydrogenase, and normal haptoglobin levels were noted. Her medical history is unremarkable for cardiovascular disease and cardiovascular risk factors. On physical examination, she had normal blood pressure at 120/70mmHg, no orthostatic hypotension, her heart rate was at 75bpm. Her cardiac auscultation revealed regular rhythm, a soft grade 1/6 systolic murmur at the apex. She had no signs of heart failure. There was no murmur at auscultation of carotids. No splenomegaly or hepatomegaly was noted. Her platelet count was low at 18,000/mm^3^, hemoglobin level at 13.2 g/dl, renal and hepatic function were normal. C-reactive protein level was normal at 9 mg/l. Electrocardiography (ECG) showed a regular sinus rhythm, without arrhythmia or repolarization abnormalities. On Holter ECG monitoring, short bursts of supraventricular premature beats were registered. Transthoracic echocardiography showed (Figure 1) an oval-shaped mass in the left atrium. The mass is lobular, non-homogenous, appended to the inter-atrial septum, in motion, pushing on the anterior mitral leaflet without prolapsing into the mitral valve. There was mild mitral regurgitation. The size and function of both ventricles were normal.

**Figure 2:**
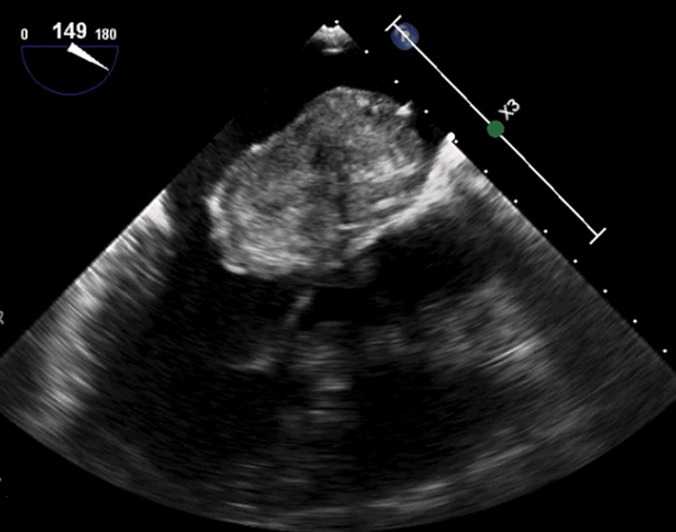
left atrial mass on 2D transoesophageal echocardiography

**Figure 3:**
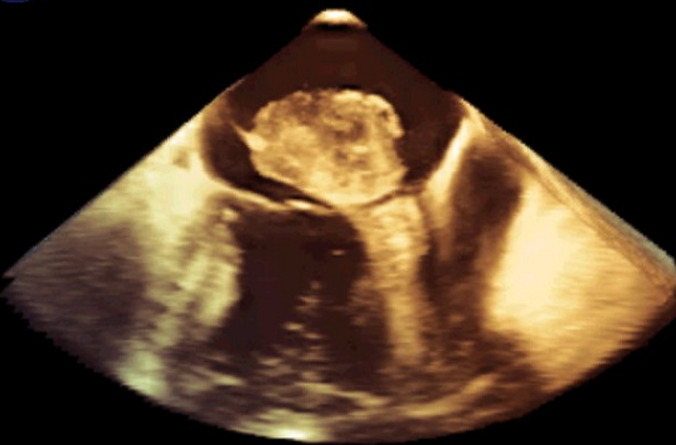
left atrial mass on 3D transoesophageal echocardiographraphy

**Figure 4:**
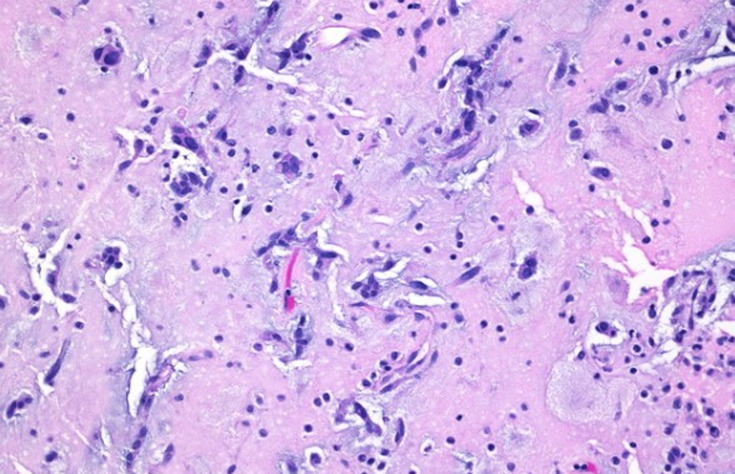
areas of stellate-shaped and fusiform cells on a myxoid background

**Figure 5:**
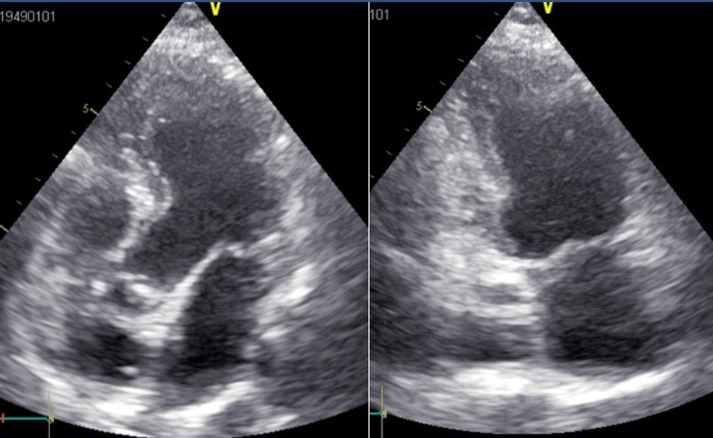
post-operative transthoracic echocardiography

On transesophageal echocardiography morphological features (Figure 2, Figure 3) of the mass were consistent with myxoma: the mass measuring 53/32, was shaped like a tennis racquet, with poorly-defined edged evoking its friable aspect, it has a heterogeneous appearance with hypoechoic areas probably related to focal necrosis, it is attached to the interatrial septum by several filamentous attachments allowing its high mobility. The mass rests on the anterior leaflet of the mitral valve, which throws it high as it closes, and it prolapses indeed sometimes into the valve presenting its closure. Cardiac magnetic resonance imaging confirmed the echocardiographic findings. The patient underwent surgery by an approach using median sternotomy and cardiopulmonary bypass, with perfusion of platelet concentrates before and during surgery, surgical exploration confirmed echocardiographic findings and macroscopic aspect of the tumor evoked myxoma. Total excision of tumor including the pedicle was performed. Pathology confirmed the diagnosis of cardiac myxoma (Figure 4). After a postoperative stay in unit care and careful monitoring of bleeding, the patient was discharged and has been doing well. Two weeks postoperatively, the patient was asymptomatic, with a normal cardiovascular exam. Platelet count was gradually increased to reach 95-103/µl after 6 months postoperatively, echocardiography showed vacuity of the left atrium, with normal mitral valve leaflets and a mild regurgitation (Figure 5).

## Discussion

Myxomas represent 0.25% of all heart diseases, of the primary cardiac tumors, approximately 80% are considered benign, and atrial myxomas account for approximately half of the benign tumors. Epidemiologically, myxomas frequently occur in middle age, with a mean age of 53.1 ± 13.5 years [1]. They show a female predominance with a sex ratio of 3: 1. Diagnosis is often difficult due to the wide array of presenting symptoms, clinical manifestations of cardiac myxoma are various without defining characteristics. A wide spectrum of clinical manifestations ranging from asymptomatic forms, identified erratically, to severe ones with complications involving life-threatening prognosis was noted [2]. Syncope is rare, reported in 1% of cases. Our patient had recurrent episodes of fainting and syncope that may be due to interposition of the myxoma between mitral leaflets with transient interruption of left ventricular filling [2]. In this context of syncope myxoma is often misdiagnosed, in our patient, the delay to diagnosis was prolonged, she had fainting until six months and thrombocytopenia until five years. Lee *et al*. [1] reported a patient with myxoma with a time interval between symptoms and diagnosis of five years. The diagnosis of a cardiac tumor is facilitated by echocardiogram. Chest computed tomography (chest-CT) scan and cardiac magnetic resonance imaging (CMR) provide also complementary information on myxomas, however, these investigations should be reserved for cases in which the diagnosis or characterization of the tumor remains unclear after an echocardiographic evaluation [3]. Our patient had both echocardiography and CMR, the data of which are compatible with the diagnosis of myxoma.

One of the specificities of this case report is the association of thrombocytopenia with cardiac myxoma. An increase in platelet count after excision of myxoma was noted. In our patient, both thrombotic thrombocytopenic purpura and idiopathic thrombocytopenic purpura have been ruled out since there was no response to steroid therapy and there was no evidence of end-organ damage and hemolytic anemia. Thrombocytopenia due to impaired production was ruled out since bone marrow yielded increased megakaryocytes. Normal lactate dehydrogenase and normal haptoglobin levels ruled out hemolysis. The absence of anemia, normal white blood cells count and normal immunologic tests excluded Evan syndrome. Autoimmune thrombocytopenia was excluded as there was no response to steroids given for many years. To the best of our knowledge, only seven cases of cardiac myxoma associated with thrombocytopenia were reported [2-8]. Table 1 summarizes clinical and biological findings in these reported cases. Thrombocytopenia has very rarely been reported in association with cardiac tumors, either benign or malignant. Cardiac tumor-associated thrombocytopenia is often associated with other hematologic disorders such as anemia or erythrocytosis as well, however, in our case thrombocytopenia was an isolated finding. The mechanism by which intracardiac tumor leads to thrombocytopenia remains unclear, although it has been postulated that abnormal mechanical shear stress, caused by tumor-induced flow obstruction may be responsible for the increase in the breakdown of platelet [9] This fact is supported by improvement in platelet count after mere removal of the cardiac mass as reported in few studies andin our patient [10]. The mechanism of thrombocytopenia in patients with myxomas is unclear. Nakamura *et al*. [8] reported a case of thrombocytopenia recovering after the resection of cardiac myxoma, and an immunohistochemical study of the resected tumor showed that the myxoma expressed CD31, also known as platelet endothelial cell adhesion molecule-1, which is known to influence change in platelets. It was also thought that the myxoma exhibiting microscopic bleeding, thrombosis, and hemosiderosis, might lead to the consumption of platelets. Another explanation, interleukin (IL6), a proinflammatory cytokine involved in many autoimmune diseases, and its overproduction by cardiac myxomas can trigger an autoimmune loop eventually leading to immune thrombocytopenia [7]. In our patient, a possible link between thrombocytopenia and myxoma was evoked because of the rise of platelet count after myxoma excision.

**Table 1:** clinical and biological findings in reported cases and in our patient

Author/year	Age/sex	Localisation of the myxoma	Clinical presentation	Platelet count before surgery	Hematologic and immunological disturbance associated to thrombocytopenia	Recovery of thrombocytopenia after surgery/platelet count
**Vuopio 1966**	58/f	Left atrium	Dyspnea palpitation severe dizziness	6-34 10^3^/μl	Anemia (hb:7.4 g/100ml)	No surgery died
**Kucharski 2003**	47/m	Left atrium	Dyspnea heart failure	thrombocytopenia	Disseminated intravascular coagulation	No surgery
**Crawford 1978**	12/f	Right atrium	fatigue	125 10^3^/μl	Hemolytic anemia	Yes/365 10^3^/μl
**Gould 1978**	na	Right atrium		na	na	na
**Burns 1982**	44/f	Right atrium	Exertional dizzines without syncope	58 10^3^/μl	Erythrocytosis hb:17 g/100ml hematocrite:50%	Yes/248 10^3^/μl [6 month]
**Durand 2019**	30/m	Right atrium	Sudden dyspnea and syncope spontaneous bruising	50 10^3^/μl	Antinuclear/antiphospholid antibodies/lupus anticoagulant positivity	Yes/rise of platelet count and remained within normal range
**Nakamura 2019**	82/m	Left atrium	Anorexia	23 10^3^/μl	Eleveted iga and platelet -associated igg (paiga)	Yes/166 10^3^/μl decreased level of paiga
**Our patient**	72/f	Left atrium	Fainting syncope	18 10^3^/μl	Isolated thrombocytopenia	Yes/95 10^3^/μl

## Conclusion

The originality of this report lies in the association of chronic thrombocytopenia with cardiac myxoma. The increase of platelet count obtained after surgical excision of the cardiac myxoma suggests a possible physiopathological link between these two entities. Another particularity of our case is the clinical presentation. Cardiac myxomas and syncope is a very rare manifestation, myxoma should be considered in the differential diagnosis of unexplained syncope.
